# A Biphasic and Brain-Region Selective Down-Regulation of Cyclic Adenosine Monophosphate Concentrations Supports Object Recognition in the Rat

**DOI:** 10.1371/journal.pone.0032244

**Published:** 2012-02-16

**Authors:** Maïte Hotte, François Dauphin, Thomas Freret, Michel Boulouard, Guenaëlle Levallet

**Affiliations:** 1 Université de Caen Basse–Normandie, Groupe Mémoire et Plasticité comportementale (GMPc), EA4259, IFR 146, Caen, France; 2 Université de Rouen, NeoVasc, EA 4309, IFRMP23, IHURBM, Rouen, France; 3 CHU de Caen, Service d'Anatomie Pathologie, Caen, France; University of Insubria, Italy

## Abstract

**Background:**

We aimed to further understand the relationship between cAMP concentration and mnesic performance.

**Methods and Findings:**

Rats were injected with milrinone (PDE3 inhibitor, 0.3 mg/kg, i.p.), rolipram (PDE4 inhibitor, 0.3 mg/kg, i.p.) and/or the selective 5-HT4R agonist RS 67333 (1 mg/kg, i.p.) before testing in the object recognition paradigm. Cyclic AMP concentrations were measured in brain structures linked to episodic-like memory (i.e. hippocampus, prefrontal and perirhinal cortices) before or after either the sample or the testing phase. Except in the hippocampus of rolipram treated-rats, all treatment increased cAMP levels in each brain sub-region studied before the sample phase. After the sample phase, cAMP levels were significantly increased in hippocampus (1.8 fold), prefrontal (1.3 fold) and perirhinal (1.3 fold) cortices from controls rat while decreased in prefrontal cortex (∼0.83 to 0.62 fold) from drug-treated rats (except for milrinone+RS 67333 treatment). After the testing phase, cAMP concentrations were still increased in both the hippocampus (2.76 fold) and the perirhinal cortex (2.1 fold) from controls animals. Minor increase were reported in hippocampus and perirhinal cortex from both rolipram (respectively, 1.44 fold and 1.70 fold) and milrinone (respectively 1.46 fold and 1.56 fold)-treated rat. Following the paradigm, cAMP levels were significantly lower in the hippocampus, prefrontal and perirhinal cortices from drug-treated rat when compared to controls animals, however, only drug-treated rats spent longer time exploring the novel object during the testing phase (inter-phase interval of 4 h).

**Conclusions:**

Our results strongly suggest that a “pre-sample” early increase in cAMP levels followed by a specific lowering of cAMP concentrations in each brain sub-region linked to the object recognition paradigm support learning efficacy after a middle-term delay.

## Introduction

Most modern theories of learning and memory postulate that memory processes require cyclic adenosine monophosphate (cAMP) synthesis [1]; however, there is little evidence concerning the mechanisms by which memory affects adenylyl cyclase activity (cAMP synthesis) and/or phosphodiesterase (PDE) activity (cAMP degradation). Literature reports indicate that activation of the cAMP-PKA pathway cascade by memory processes triggers activation of transcription factors such as CREB [2], leading to neural processes that underlie learning and memory [1], [3]–[5]. Consequently, several studies argue that artificial cAMP-PKA cascade activation through intra-hippocampal infusion of 8Br-cAMP, adenylyl cyclase or PKA activation improves memory performance [4], [6]–[9] whereas pharmacological inhibition of PKA disrupts hippocampal long term potentiation and hippocampus-based long-term memory [6], [10], [11]. Memory efficiency seems, however, to require a restricted or selective cAMP production; high cAMP levels do not necessarily improve memory. Indeed, studies on flies and mice show that increases in adenylyl cyclase activity can result in memory deficits [12], [13]. Similarly, increasing PKA activity impairs prefrontal cortex-dependent memory in mice and expression of a constitutively active isoform of the G-protein subunit Gαs impairs mice behavioural performance in a fear-conditioning task [14]. These works clearly demonstrate the complexity of cAMP-dependent responses.

Mnesic mechanisms may be investigated through the use of an object recognition memory task, a one-phase task based on spontaneous activity and the natural preference that rodents display to explore a novel object rather than a familiar one [15]. With this paradigm, memory performances were demonstrated to be enhanced by the activation of serotonin 5-HT4 receptors (5-HT4R) [16]–[19], receptors that have been also demonstrated to be implicated in short- and long-term memory processes in laboratory animals [20]–[30] (for review see [31]). Activation of 5-HT4R, positively coupled to adenylyl cyclase, induce increases in cAMP concentrations that can be regulated by activation of cAMP phosphodiesterases (PDE) isoforms from families 1, 2, 3, and 4 [18]. Involvement of PDE4 inhibitors in working and reference memory [5], [32]–[34] has already been well investigated through the use of rolipram, a selective PDE4 inhibitor. In fact, several studies have already reported a positive effect of PDE4 inhibition on spatial memory [3], [5], [33]–[37], inhibitory avoidance learning [5], [33], [38], contextual fear conditioning [4], [39], and object recognition [40]–[42]. The PDE4 isoenzymes are encoded by four independent genes (Pde4a to Pde4d), which generate more than 25 splice variants [43], [44]. Each splice variant exhibits unique properties leading to specific control of cAMP levels [45], [46]. Few studies deal with the involvement of each PDE4 isoforms in memory performance as few studies have also investigated the effect of others cAMP-PDE families on memory performance, especially in the object recognition task [42], [47]. Thus, from the literature, little is known about the behavioural consequence and especially memory performance following PDE4 [18], PDE3 [48] or PDE2 [47] inhibition although these PDE families accounts for a major part of the total cAMP-PDE-hydrolysing activities in the hippocampus, the prefrontal and perirhinal cortices, brain structures involved in recognition memory [18].

A way to enhance cAMP signaling and consequently influence the pathways involved in object recognition (episodic-like) memory, is to stimulate 5-HT4Rs and/or inhibit PDE enzymes, especially PDE3 and 4 [18]. Here, we further characterize the respective role of PDE3 and PDE4 in the processes of recognition memory and assayed the relationship between cAMP concentrations and mnesic performance. With this aim, we injected rats before the acquisition phase, with milrinone (a selective PDE3 inhibitor currently used in heart failure studies [49], with a half-life of 1.5–2.3 h [50], [51], 0.3 mg/kg), or rolipram (a selective PDE4 inhibitor with good brain penetration and a relatively short half-life of 1–3 h [52], 0.3 mg/kg) both alone and in combination or not with the selective 5-HT4R agonist RS 67333 (1 mg/kg) [17], [18]. The half-life of RS 67333 in the rat is not reported in the literature, but the data from behavioral studies [17], [18] suggest that this is comparable (1–3 hours) to those of the two PDE inhibitors.

Before or after either the sample or the testing phase, cAMP concentrations were measured in the hippocampus, prefrontal and perirhinal cortices. We show that a “pre-sample” early increase in cAMP levels followed by a specific lowering of cAMP concentrations in each brain sub-region linked to the object recognition paradigm support learning efficacy after a middle-term delay. Following the different treatments and at the end of the testing trial, we also examined in these brain sub-regions i) the PDE activities to validate efficiency of PDE3 or PDE4 inhibition by their respective inhibitor and ii) the phosphoprotein phosphatase (PP) type 2 activities since cAMP concentrations have been shown to be transiently up- or down-regulated by PP2A activation in various cell types [53]–[55]. In fact, as cAMP-PDE limit excessive cAMP production by catalysing its hydrolysis; PP1 and PP2 (accounting for more than 90% of total phosphatase activity in brain [56]) limit PDE-induced excessive catabolism of cAMP by reversing PP2A phosphorylation of phosphorylated PPE3B [57] or particulate PDE4 activities [58]. We also demonstrate here, that milrinone alters type PP2 activities in anatomical structures linked to object recognition memory in rat.

## Materials and Methods

### 1. Subjects

A total of 172 adult male Sprague-Dawley rats (300–350 g, René Janvier, France) were used in these experiments. Rats were housed in groups of three in a temperature controlled room under a 12L∶12D cycle (lights on at 8:00 pm), with food and water provided ad libitum. All procedures were performed in conformity with National (JO 887–848) and European (86/609/EEC) legislations on animal experimentation. Behavioural procedures received approval from the Ethics Committee for Animal Experimentation of Normandy (Approval number 1009-01).

### 2. Behavioural experiments

#### 2.1 Apparatus

The apparatus consisted of an open-box (100×100×60 cm) made of wood with the inside painted in black. The objects to be discriminated were made of plastic, or glass (all 5 cm height) and were available in four copies. The objects were fixed (Patafix) on the floor in the box, to ensure that they could not be displaced by the rats.

#### 2.2 Handling and habituation

Rats were handled daily for one week prior to the study and then habituated to the apparatus and the test room. The first two days, rats were put together as a group of 3 to explore the empty arena for 10 min. On the third day, rats were put individually in the empty box for 3 min and the next two days, in the presence of an object that will not be used for the experimental task. Testing began on day 6.

#### 2.3 Object recognition task

Animals were tested in the object recognition task as described previously [15], [18]. The test session consisted of two phases with a duration of 3 min each on day 6. During the sample phase, each rat was placed in the box with two identical objects (placed close to the corners). After a delay of 4 h, during which the animal returned to its cage and both objects were replaced (one by its identical copy, the other by a new object in the same locations), the rat was returned to the box (testing phase). From rat to rat, the role (familiar or new object) as well as the relative position of the two objects were counterbalanced and randomly permuted.

The number of animals in each group was: saline-treated (n = 32), RS 67333 1 mg/kg (n = 32), milrinone 0.3 mg/kg (n = 27), milrinone+RS 67333 (n = 27), rolipram 0.3 mg/kg (n = 27), rolipram+RS 67333 (n = 27).

### 3. Drugs and drug administration

In all experiments, each rat was given an i.p injection of either saline (NaCl 0.9%) or RS 67333 (1 mg/kg) 30 minutes prior to the sample phase as previously described [18]. We have not tested other steps of memorization or lower doses of RS 67333 because i) RS6733-induced enhancement was reported only for the acquisition phase of information processing and ii) doses of 0.001 or 0.01 mg/kg were ineffective to enhance recognition memory [16], [17]. Milrinone (PDE3 inhibitor) or rolipram (PDE4 inhibitor) was injected each at the dose of 0.3 mg/kg i.p. 45 min prior to the sample phase. Higher doses of PDE inhibitors were not tested since at high dosage, milrinone can have vasodilatory and arrhythmogenic effects [59] and rolipram can have sedative side-effects [60], [61]. Efficiency of PDE3 or PDE4 inhibition was confirmed by specific PDE3 or 4 activity measures in hippocampus, prefrontal and perirhinal cortices at the end of the behavioural task. Moreover, since object recognition performance can only be determined if the animals show sufficient exploration [62], we concomitantly evaluated the exploration levels of the animals. In our experiment, a dose of 0.3 mg/kg rolipram or 0.3 mg/kg milrinone, given 45 min before the sample phase, resulted in a substantial decrease in locomotor activity but not in exploratory behaviour. A total of 148 rats were used to perform the object recognition task (tested animals), and 24 rats received the different injections, without being subjected to the behavioural task (untested animals).

### 4. Biochemical analysis

#### 4.1 Cyclic AMP measurement

Cyclic AMP extraction was performed according to a procedure adapted from Rodriguez [63]. Immediately after the testing phase or at the corresponding delay following the different injections (for untested animals), rats were subjected to euthanasia by decapitation without prior anesthesia. Intact brains were dissected on ice into prefrontal cortex, perirhinal cortex and hippocampus, taken systematically in this order and in less than 3 min following euthanasia. Brain sub-regions were rinsed with cold saline and dried. Each sub-region was homogenized in cold 100% ethanol in an ice bath and the homogenate centrifuged at 20,000 g for 15 min. The supernatant was recovered and, the pellet resuspended in 1 ml of 2∶1 ethanol∶water solution and centrifuged as before. The combined supernatants were evaporated to dryness in a 60°C bath under a stream of nitrogen gas. The final residue was dissolved in 0.5 ml of assay buffer (0.05 M sodium acetate, pH 5.8, containing sodium azide). Cyclic AMP levels were determined with a radioimmunoassay (Amersham). This assay measures the competitive binding of ^3^H-labeled cAMP to a cAMP-specific antibody.

#### 4.2 Preparation of rat brain membranes and soluble fractions

Sub-cellular fractionation of the brain regions was also performed immediately after euthanasia as detailed previously [18]. Briefly, each cerebral tissue was placed, immediately upon isolation, into ice-cold homogenization buffer (20 mMTris-HCl pH 7.2, 1 mM EDTA, 250 mM sucrose, supplemented with 0.1 mMphenylmethanesulfonyl fluoride, 2 mMbenzamidin, and a mixture of antiproteases (antipain, aprotinin, leupeptin, pepstatin A) at a final concentration of 1 microg/ml), homogenized by several passages through 25-G needle. Homogenates were centrifuged at 1,000 g, 4°C for 5 min and the supernatants decanted and centrifuged at 100,000 g, 4°C for 1 h. Each supernatant (soluble fraction) and the respective pellet (particulate fraction, re-suspended in ice-cold complete homogenization buffer) were then stored at −20°C. Protein content of each fraction was determined by the method of Bradford with BSA as a standard [64]. Purity of each subcellular fraction was assayed by both lactate deshydrogenase (soluble activity) and alkaline phosphatase (membrane-associated activities) as already reported [18].

#### 4.3 PDE Assay

Phosphodiesterase activities were assayed according to the two-step modified procedure of Thompson and Applemann [65] as already described [18]. To discriminate PDE2, PDE3 or PDE4 activities from other PDE activities, protein from each sample were incubated either in the absence (total PDE activities) or in the presence of specific inhibitors of each family: 20 µM erythro-9-(2-hydroxy-3-nonyl)-adenine (EHNA), 20 µM milrinone or 10 µM rolipram for PDE2, PDE3 and PDE4, respectively, according to their respective IC50 described elsewhere [18]. Differences between total and selective inhibitor-insensitive PDE activities were considered as corresponding PDE activities.

#### 4.4 Phosphatase Assay

Total PP2 activities in subcellular fractions of the different rat brain structures were determined by Serine/Threonine Phosphatase Assay (Promega, Charbonière-les-Bains, France) which used a specific substrate for PP2. Assays were conducted according to the manufacturer's procedure. Free phosphate was then quantified by a colorimetric method.

#### 4.5 SDS-PAGE Western Blot Analysis

Subcellular fraction protein from hippocampus, prefrontal and perirhinal cortices were boiled for 5 min and separated by 8% SDS-PAGE. The proteins were transferred onto a nitrocellulose membrane (1 h at 100 V and 4°C). Western blotting was then performed using an affinity-purified goat polyclonal antibody raised against a peptide that maps near the C-terminus of the human PDE4D (Santa Cruz Biotechnology). Immunoblotting with antibody that was pre-incubated with an excess of the peptide used for immunization (Santa Cruz Biotechnology) was performed as a negative control, following the instructions of the supplier. Immunoreactive bands were detected using a donkey anti-goat IgG-horseradish peroxidase (HRP) complex and an enhanced chemiluminescence (ECL) Advance Western Blotting Detection Kit (Amersham Biosciences). For β-actin detection, the blots were stripped in a stripping buffer that contained 62.5 mM Tris-HCl (pH 6.7) 2% SDS, and 100 mM β-mercaptoethanol at 58°C for 30 min, and reprobed for actin with monoclonal mouse anti-actin antibody and goat anti-mouse IgG-HRP (Calbiochem). The immunoblots were scanned on the ProXPRESS Proteomic Imaging System (Perkin Elmer Life Science, Boston, MA) and analyzed with the TotalLab Image Analysis software (Nonlinear Dynamics Ltd., Newcastle, UK).

### 5. Data scoring and analysis

#### 5.1 Behavioural analysis

The experimenter sat in front of the box. Total time spent exploring each object in both the sample and the testing phases were recorded. Exploration of an object was defined as follows: directing the nose to the object at a distance <2 cm. Overall exploration times across phases were analyzed by a two-way ANOVA (phase and treatment as factors) with repeated measures. For testing phase data, exploration of each object was analyzed using a two-way repeated-measurements ANOVA with object and treatment as factors. When appropriates, post-hoc testing was performed using Fisher's least significant difference (LSD) test. We calculated discrimination indexes as D1, which is the difference in time spent exploring the two objects in testing trial (i.e. time with novel object minus time with familiar object); and D2, the discrimination ratio, which is the difference in exploration time (D1) expressed as a ratio of the total time spent exploring the two objects in the testing trail (e.g. novel-familiar/novel+familiar). This ratio makes it possible to adjust for individual or group differences in the total amount of exploration time. Comparisons were made using one-way ANOVA with treatment as factor and post-hoc testing was performed using Fisher's least significant difference (LSD) test.

Locomotor activity was measured during the test session through videotaping. The arena was divided into 9 squares (32×32 cm). During each phase, the number of entries in each square was measured. Analysis was performed using two-way repeated-measurements ANOVA with entry and treatment as factors, followed by Fisher's LSD test when necessary.

#### 5.2 Biochemical analysis

After construction of a standard curve, cAMP levels were determined directly from the counts (in duplicate for each brain region of each animal) in nanomoles per milligram of tissue wet weight. PDE activities (in triplicates) were expressed in pmol of cAMP hydrolyzed per min and mg of protein. PP2 activities were expressed as nmol of phosphate released per min.

Statistical differences were determined through non-parametric tests adapted to small size data (Friedman and Kruskal-Wallis, followed by a post-hoc Mann-Whitney U-test; Sigma Stat software SPSS Inc, Chicago, IL).

## Results

### Hippocampus, prefrontal and perirhinal cortices exhibit different patterns of particulate PDE4D isoforms

**Figure 1:**
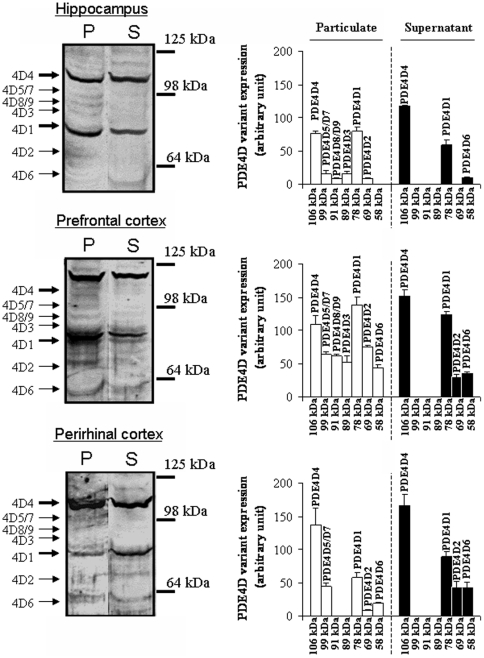
Expression of PDE4D proteins in the particulate and soluble fractions of rat hippocampus, prefrontal and perirhinal cortices. Particulate and soluble fractions from the rat hippocampus, the prefrontal cortex and perirhinal cortex were isolated and proteins extracted as described in Materials and Methods section. The left panel shows representative immunoblots of particulate (25 µg) and soluble (25 µg) protein fractions probed with goat polyclonal human anti-PDE4D antibody in the hippocampus, prefrontal and perirhinal cortices. Arrowheads indicate the molecular weights of the immunoreactive proteins. The right panel shows quantification; the intensities of the immunoreactive bands in the particulate and soluble fractions from hippocampus, prefrontal and perirhinal cortices were determined and normalized to those of actin. The densitometry values are the mean ± SEM (n = 3).

Cyclic AMP-PDE was assayed in subcellular fraction from hippocampus, prefrontal and perirhinal cortices. Here, we confirmed our previous work [18] reporting that the total cAMP-PDE-hydrolysing activities of the particulate fraction from hippocampus, prefrontal and perirhinal cortices are mainly composed by PDE3 (38,2%, 34,8% and 43,4% respectively in hippocampus, prefrontal and perirhinal cortices) and PDE4 (26,7%, 43,4% and 21,4% respectively in hippocampus, prefrontal and perirhinal cortices) (data not shown). Since each PDE4D isoform plays specific roles on the cAMP concentration feedback [45], [46], we furthermore characterized by western blotting the pattern of PDE4D isoforms present in these brain sub-region (**Fig. 1**). We demonstrated that PDE4D protein expression differed according to the subcellular fraction and the brain sub-region. In the particulate fraction from prefrontal cortex, the presence of nine immunoreactive proteins suggests that all nine PDE4D isoforms (i.e. PDE4D1 to PDE4D9) are expressed whereas particulate fraction of hippocampus did not exhibit PDE4D6 and of perirhinal cortex neither particulate PDE4D8/9 nor particulate PDE4D3. Nevertheless, in the particulate fraction from hippocampus, prefrontal or perirhinal cortices, both the short PDE4D1 and the long PDE4D4 isoforms are the mainly PDE4D isoforms expressed. Finally, PDE4D1, PDE4D2, PDE4D4 and PDE4D6 were the isoforms revealed in the soluble fraction from prefrontal or perirhinal cortices. A similar panel of PDE4D isoform was revealed in the soluble fraction from the hippocampus, except that no immunoreactive band matches with PDE4D2. As reflected by the densitometric analyses (right panel of **Fig. 1**), the patterns of the putative PDE4D isoforms did not display any significant structure-related differences.

### RS 67333 enhances particulate PDE3 activity from the perirhinal cortex in rat

**Figure 2:**
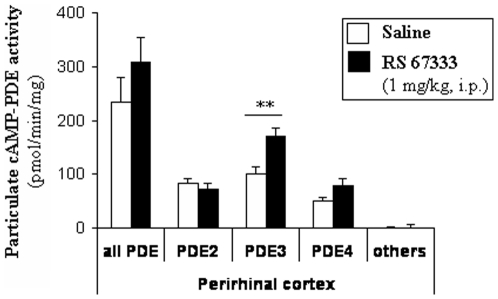
Effect of RS 67333 (1 mg/kg, i.p.) on PDE activities in the perirhinal cortex from rats performing the object recognition task with a 4-h delay. Rats were injected with saline or the 5-HT4 receptor agonist RS 67333 (1 mg/kg, i.p.), 30 minutes before exposure to the sample trial of he object recognition task. Immediately after the testing trial, particulate fractions from the hippocampus, prefrontal and perirhinal cortices were isolated and assayed for EHNA- (20 µM), milrinone- (20 µM) and rolipram- (10 µM) sensitive PDE activities, respective inhibitors of PDE2, PDE3 and PDE4 families. PDE inhibitor-sensitive and –insensitive PDE activities were each expressed as pmolcAMPhydrolysed/min/mg protein. Results are means ± SEM of four independent subcellular fractionations performed in triplicate. Within each subcellular compartment, * indicates a significant difference of PDE activity as compared with saline treatment within a family of PDE activity (PDE2, 3, 4 or other PDE) (**, *P*<0.01, ANOVA followed by Fisher's LSD test).

To identify which cAMP-PDE family support the rolipram-insensitive PDE activities increased in the perirhinal cortex following the selective activation of 5-HT4R (RS 67333; [18]), rats were injected with a saline solution or RS 67333 (1 mg/kg, i.p.) before the object recognition paradigm (inter-phase interval of 4 h). Immediately after the testing phase, rats were subjected to euthanasia and PDE activities were assayed. As shown in **Fig. 2**, RS 67333 elevated PDE3 activities by 71% (P<0.01) in the perirhinal cortex lightening this family as a key regulator of cAMP concentration in this structure linked to object recognition. No variation was measured in the supernatant fraction (data not shown).

### 5-HT4 receptor stimulation, PDE3- or PDE4-inhibition improve familiar object recognition after a 4-h delay in rat

Rats were then injected with milrinone (PDE3 inhibitor, 0.3 mg/kg, i.p.), rolipram (PDE4 inhibitor, 0.3 mg/kg, i.p.) and/or the selective 5-HT4R agonist RS 67333 (1 mg/kg, i.p.) before the object recognition paradigm sample phase.

**Figure 3:**
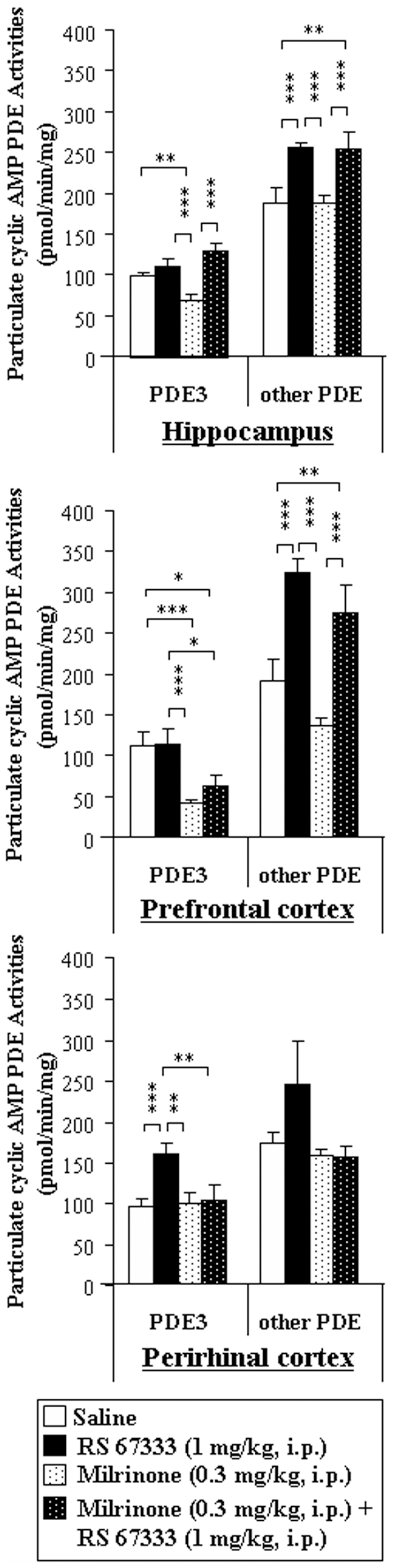
Milrinone (0.3 mg/kg, i.p.) specifically inhibits PDE3 activities in hippocampus, prefrontal cortex and perirhinal cortex from rats. Rats were injected with the PDE3 inhibitor (milrinone, 0.3 mg/kg, i.p.) and then with saline or the 5-HT4 receptor agonist (RS 67333, 1 mg/kg, i.p.), respectively 45 minutes and 30 minutes before the sample phase of the object recognition task. Immediately after the testing phase, both particulate and soluble fractions from the hippocampus, the prefrontal cortex and perirhinal cortex were isolated and particulate fraction was assayed for milrinone (20 µM)-sensitive PDE activities. Milrinone-sensitive and –insensitive PDE activities were expressed as pmolcAMPhydrolysed/min/mg protein. Results are means ± SEM of four independent subcellular fractionations performed in triplicate. Within each subcellular compartment, * indicated significant differences of PDE activity as compared with other treatment within a type of PDE activity (PDE3 or other PDE) (*, P<0.05, **, P<0.01, ***, P<0.01, ANOVA followed by Fisher's LSD test).

**Figure 4:**
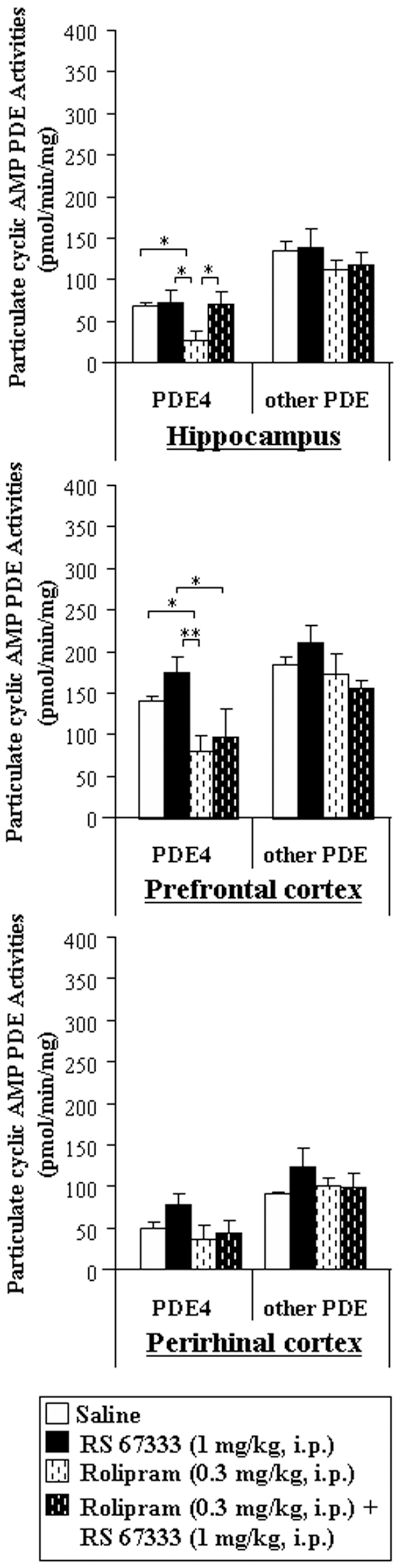
Rolipram (0.3 mg/kg, i.p.) specifically inhibits PDE4 activities in hippocampus, prefrontal cortex and perirhinal cortex from rats. Rats were injected with the PDE4 inhibitor (rolipram, 0.3 mg/kg), and then with saline or the 5-HT4 receptor agonist (RS 67333, 1 mg/kg), respectively 45 minutes and 30 minutes before the sample phase of the object recognition task. Immediately after the testing phase, both particulate and soluble fractions from the hippocampus, the prefrontal cortex and perirhinal cortex were isolated and the particulate fraction was assayed for rolipram (10 µM)-sensitive PDE activities. Rolipram-sensitive and –insensitive PDE activities were expressed as pmolcAMPhydrolysed/min/mg protein. Results are means ± SEM of four independent subcellular fractionations performed in triplicate. Within each subcellular compartment, * indicated significant differences of PDE activity as compared with other treatment within a type of PDE activity (PDE4 or other PDE) (*, P<0.05, **, P<0.01, ANOVA followed by Fisher's LSD test).

We first validate the efficiency of the treatments with PDE inhibitors; rats were immediately subjected to euthanasia after the testing phase, and PDE activities from the hippocampus, prefrontal and perirhinal cortices, were assessed. We especially measured PDE3 activity for milrinone-treated animals (**Fig. 3**) and PDE4 activity for rolipram-treated animals (**Fig. 4**). Concerning the measurements of particulate PDE3 activities in milrinone-treated rats (**Fig. 3**), we showed that PDE3 activity was inhibited in the hippocampus (−30%, P<0.01) and prefrontal cortex (−63%, P<0.001), but not in the perirhinal cortex, when compared to saline-treated rats. However, pre-treatment of rats by milrinone before RS 67333 prevented the RS 67333-induced increase in particulate PDE3 in the perirhinal cortex. Finally, milrinone did not affect significantly cAMP-PDE activities supported by other families than PDE3. As illustrated in **Fig. 4**, in rolipram-treated rats, particulate PDE4 activity was lower than in saline-treated rats, in the hippocampus (−60%, P<0.05) and the prefrontal (−42%, P<0.05) cortex, while tend to be lower in the perirhinal cortex (−26%). Similar decrease when compared to RS 67333-treated group was also observed for rolipram+RS 67333-treated animals in the prefrontal cortex (−42%, P<0.05) and, despite no significant, in the perirhinal cortex (−47%).

As shown in **Table 1**, all rats spent a similar total time exploring both objects during either the sample or the testing phase after a 4-h delay (P>0.05). Comparison of locomotor activities revealed an overall significant effect of treatment during the sample phase (F (5, 112) = 9.25, P<0.001). Post hoc analyses showed that rolipram- or rolipram+RS 67333-treated rats had a smaller number of entries compared to saline-treated (respectively P<0.001, P<0.01), RS 67333-treated (P<0.001), milrinone-treated (respectively P<0.001, P<0.05), milrinone+RS 67333- treated animals (respectively P<0.001, P<0.01) (**Table 2**). Finally, we found that milrinone-treated animals explore less than RS 67333-treated ones (P<0.01). Analysis of the testing phase revealed no significant treatment effect on the exploratory behaviour.

**Table 1:** Time of exploration of objects measured during the sample and the testing trails in the object recognition task.

	Time exploring objects (in s, mean ±SEM)	
	Sample	Testing
**Saline** (n = 32)	31.7±2.9	29.4±2.4
**RS 67333 (1 mg/kg, i.p.)** (n = 32)	33.1±3.1	34.6±2.5
**Rolipram (0.3 mg/kg, i.p.)** (n = 27)	29.8±3.5	32.6±2.4
**Rolipram (0.3 mg/kg, i.p.)+RS 67333 (1 mg/kg, i.p.)** (n = 27)	32.7±3.9	36.9±3.4
**Milrinone (0.3 mg/kg, i.p.)** (n = 27)	29.8±3.2	31.7±3.3
**Milrinone (0.3 mg/kg, i.p.)+RS 67333 (1 mg/kg, i.p.)** (n = 27)	27.3±2.7	26.7±2.4

**Table 2:** Locomotor activity measured during the sample and the testing trails in the object recognition task.

	Total number of entries (mean ±SEM)	
	Sample	Testing
**Saline** (n = 32)	50.8±2.1	48.9±3.0
**RS 67333 (1 mg/kg, i.p.)** (n = 32)	54.9±1.4	51.8±2.6
**Rolipram (0.3 mg/kg, i.p.)** (n = 27)	36.2±2.6	49.9±3.3
**Rolipram (0.3 mg/kg, i.p.)+RS 67333 (1 mg/kg, i.p.)** (n = 27)	37.6±3.4	53.3±3.3
**Milrinone (0.3 mg/kg, i.p.)** (n = 27)	46.2±2.6	46.7±3.2
**Milrinone (0.3 mg/kg, i.p.)+RS 67333 (1 mg/kg, i.p.)** (n = 27)	51.8±2.9	49.8±2.5

**Figure 5:**
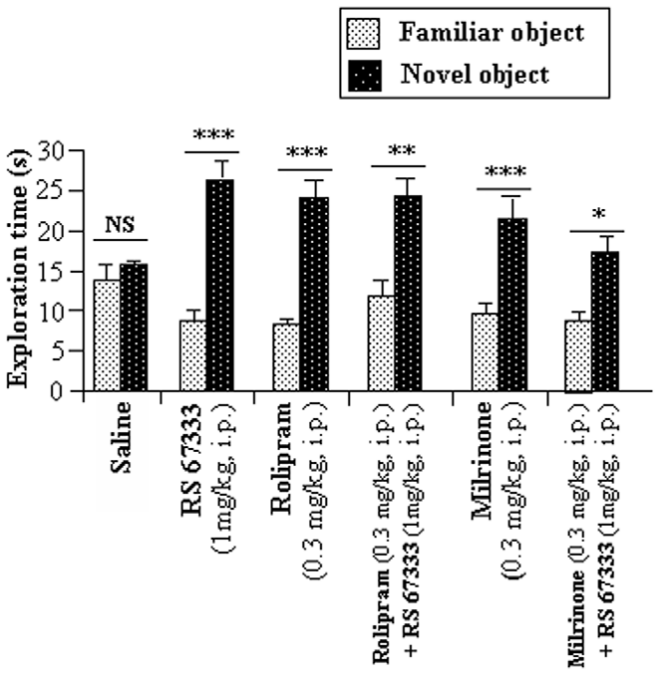
Object recognition task after a 4 h-delay in saline, rolipram- or milrinone- treated rats with or without RS 67333 co-treatment. Time of exploration of the familiar and novel objects during the testing phase of the object recognition memory task of saline (n = 32), RS 67333 (n = 32), rolipram (n = 27), rolipram+RS 67333 (n = 27), milrinone (n = 27), or milrinone+RS 67333 (n = 27)-treated rats. Rats were injected with the PDE inhibitor (0.3 mg/kg, i.p.) solution 45 minutes before the sample phase and with saline or RS 67333 (1 mg/kg, i.p.) solution 30 minutes before the sample phase. Object recognition was assayed after a 4 h-delay. Values are means in s ± SEM. NS: non significant, (*) indicates a significant difference in comparison with saline treatment. (*, P<0.05, **, P<0.01, ***, P<0.001, ANOVA followed by Fisher's PLSD test).

The repeated-measures ANOVA revealed i) for saline-treated rats, no significant difference of novel object exploration time (**Fig. 5**); ii) for drug-treated rats, both an overall significant effect of time spent exploring each object (F(1, 112 = 109.3, P<0.001) and an interaction between time exploring each object and treatment (F 5, 122) = 5.4, P<0.001). Post hoc analyses showed that all drug-treated rats significantly spent more time exploring the novel object, when compared to saline-treated rats (RS 67333-treated, P<0.001, rolipram-treated, P<0.001; rolipram+RS 67333-treated, P<0.01, P<0.001; milrinone-treated, P<0.001; milrinone+RS 67333-treated, P<0.05) (**Fig. 5**). This result is also confirmed by analysis of discrimination indexes (**Table 3**). ANOVA performed on D1 and D2 showed a significant treatment effect [for both D1 and D2: (F(5, 112) = 4.5, P<0.001)]. Post-hoc analysis revealed that all treated animals had a greater discrimination index (D1) compared to saline-treated animals (RS 67333-treated (P<0.001), rolipram-treated (P<0.001), rolipram+RS 67333 treated (p<0.01), milrinone-treated (P<0.01) milrinone+RS 67333- treated animals (P<0.05). These results are also confirmed by post-hoc analysis of the discrimination ratio (D2), compared to saline-treated rats (RS 67333-treated (P<0.001), rolipram-treated (P<0.001), rolipram+RS 67333 treated (p<0.05) milrinone-treated (P<0.01) milrinone+RS 67333- treated animals (P<0.05).

**Table 3:** Value of the index measures of discrimination in the object recognition task.

	D1 (mean±SEM)	D2 (mean±SEM)
**Saline** (n = 32)	1.4±2.3	0.1±0.08
**RS 67333 (1 mg/kg, i.p.)** (n = 32)	17.2±2.7***	0.5±0.06***
**Rolipram (0.3 mg/kg, i.p.)** (n = 27)	16.4±2.2***	0.5±0.06***
**Rolipram (0.3 mg/kg, i.p.)+RS 67333 (1 mg/kg, i.p.)** (n = 27)	12.9±3.6**	0.3±0.08*
**Milrinone (0.3 mg/kg, i.p.)** (n = 27)	12.2±2.6**	0.4±0.07**
**Milrinone (0.3 mg/kg, i.p.)+RS 67333 (1 mg/kg, i.p.)** (n = 27)	8.5±2.3*	0.3±0.07*

* P<0.05,

** P<0.01,

*** P<0.001, one-way ANOVA followed by Fisher LSD test, comparison with saline-treated group.

### Familiar object recognition is associated with a “pre-sample” early increase in cAMP levels in hippocampus, prefrontal and perirhinal cortices

**Figure 6:**
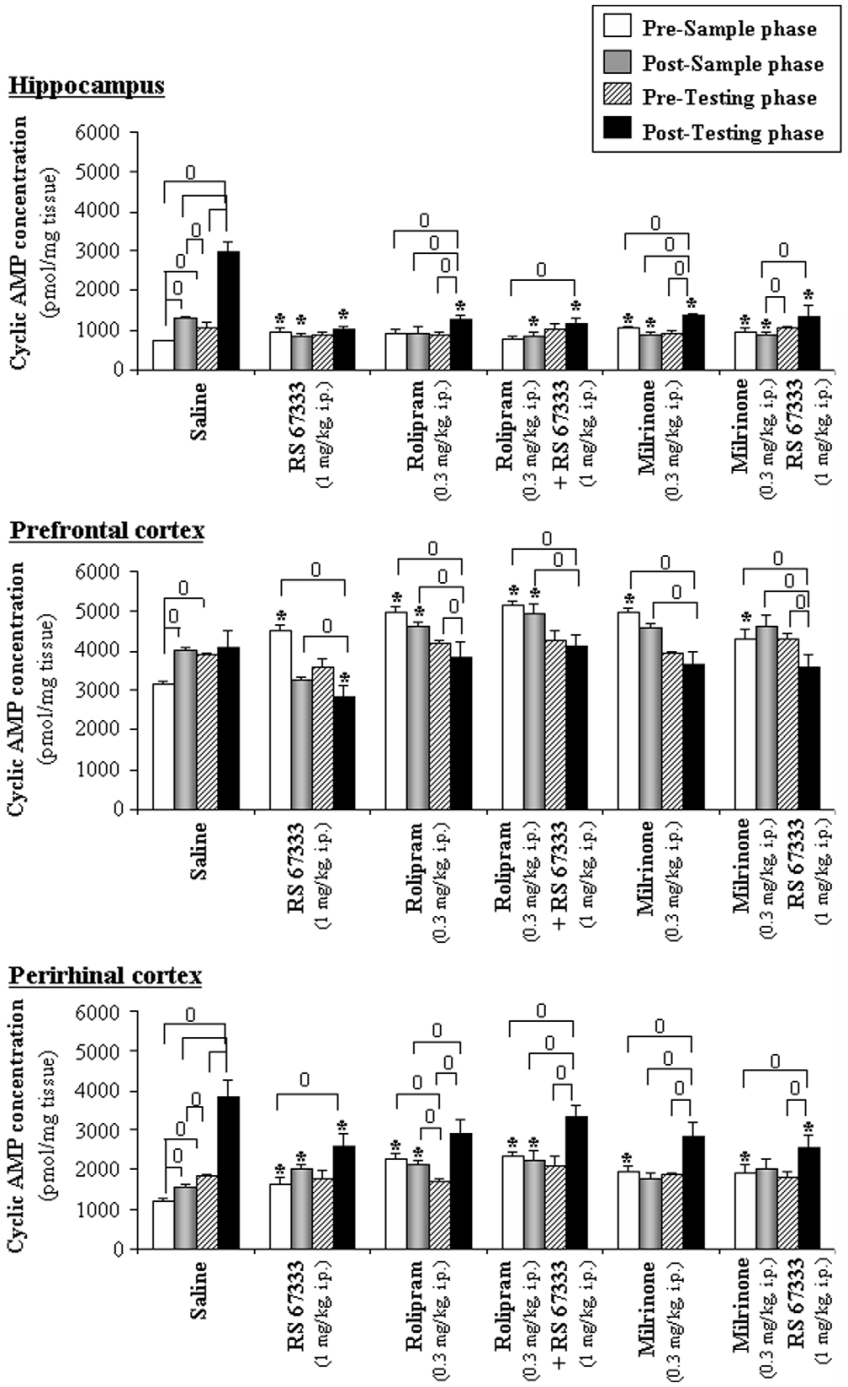
Rolipram and/or RS 67333 induce a biphasic modulation of cAMP concentrations in the hippocampus, prefrontal and perirhinal cortices of rats performing an object recognition task with a 4-h delay. Rats were injected with the inhibitor of PDE4 (rolipram, 0.3 mg/kg, i.p.) and then with saline or the 5-HT4 receptor agonist (RS 67333, 1 mg/kg, i.p.), respectively 45 minutes and 30 minutes before to the sample phase of the object recognition task. Rats were euthanized before or after the sample phase, or before or after the testing phase. Cyclic AMP was extracted from the hippocampus, prefrontal and perirhinal cortices and then assayed. Cyclic AMP was expressed as pmolcAMP/mg of weight tissue. Results were means ± SEM of three independent extractions performed in duplicate. (0) indicated significant differences in comparison with other steps of the paradigm in each brain sub-region, Mann-Whitney test, P<0.05. (*) indicated a significant difference in comparison with saline treatment in each brain sub-region. (0,*, P<0.05).

To further characterize the cellular mechanisms involved after a 4 h-delay, rats were subjected to euthanasia before or after the sample or the testing phase of the paradigm, and cAMP was measured in the anatomical structures linked to the object recognition task (i.e. hippocampus, prefrontal and perirhinal cortices) (**Fig. 6**).

As illustrated in **Fig. 6**, cAMP concentrations measured before the sample phase (white bars), demonstrate, except in the hippocampus of rolipram treated-rats, the efficiency of RS 67333 (1 mg/kg, i.p.), rolipram (0.3 mg/kg, i.p.) and milrinone (0.3 mg/kg, i.p., data not illustrated for more readability) to increase cAMP levels in the three brain structures studied, i.e. hippocampus (∼1.3 fold), prefrontal (∼1.5 fold) and perirhinal cortices (∼1.6 fold) when compared to saline-treated rats (Mann-Whitney test *versus* saline group, P<0.05).

### RS 67333, milrinone and rolipram treatments prevent the sample phase-induced increase in cAMP levels in the rat central nervous system

Analysis of the cAMP concentrations reveal that, in saline-treated rats, the sample phase induces an increase in cAMP levels in all brain regions studied (Fig. 6, grey bars versus white bars; ∼1.8 fold, ∼1.3 fold and ∼1.3 fold for the hippocampus, prefrontal and perirhinal cortices, respectively; Mann-Whitney test, P<0.05). Such a sample phase-induced increase could not be observed for the drug-treated animals; a tendency to a decreased level of cAMP was even noticed in the prefrontal cortex of these animals (∼0.86 to 0.62 fold according to drug treatment, Fig. 6) except those treated with milrinone+RS67333.

### Drug treatments lower cAMP levels in hippocampus, prefrontal cortex and perirhinal cortex after the object recognition test

After the testing phase (black bars, **Fig. 6**) when compared with the situation before the testing phase (hatched bar), cAMP concentrations in saline –treated rats were once more increased in both the hippocampus (2.76 fold, P<0.05) and the perirhinal cortex (2.10 fold, P<0.05) but not in the prefrontal cortex. Nevertheless cAMP concentrations in the prefrontal cortex from saline-treated animals still tend to be higher than before the sample phase (P>0.05). Thus, between the beginning and the end of the paradigm, we reported a 4.1, 1.3 and 3.2 fold increase in cAMP concentrations, respectively in the hippocampus, prefrontal cortex and perirhinal cortex from the saline group (Mann-Whitney test, respectively P<0.05; P>0.05 and P<0.05).

In the drug-treated animals, minor increases in cAMP levels (lower than in the saline-treated group) were also reported after the testing phase. Both rolipram and milrinone alone or administered before RS 67333 induced an almost 1.4 fold increase in cAMP concentrations in the hippocampus between the beginning and the end of the paradigm (P<0.05). Similarly, in the perirhinal cortex, a 1.6 fold increase of cAMP levels in the rolipram group (Fig. 6, P<0.05) and a 1.5 fold increase in the milrinone group (P<0.05) were reported after the testing phase. Finally, in the prefrontal cortex from drug-treated rats, cAMP levels still tend to decrease (∼−8 to −22% according to drug treatment).

At the end of the paradigm, cAMP concentrations from rats injected with a PDE inhibitor alone or in combination with RS 67333 were markedly lower when compared to saline-treated rats in the hippocampus (−54 to −61% according to the treatment, P<0.05), the prefrontal (−13 to −31% according to the treatment) or perirhinal cortices (−13 to −35% according to the treatment) (Mann-Whitney test *versus* saline group, P<0.05).

### Milrinone altered PP2 activities in anatomical structures linked to object recognition memory in rat

**Figure 7:**
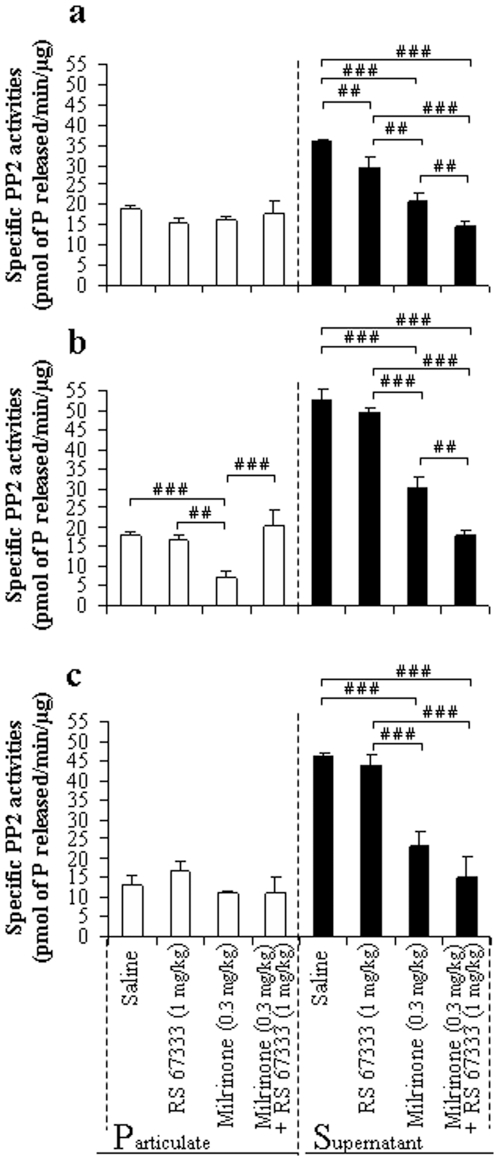
Effect of milrinone (0.3 mg/kg) on PP2 activities in hippocampus, prefrontal and perirhinal cortices from rats performing the object recognition task with a 4-h delay. Rats were injected with the inhibitor of PDE3 (milrinone, 0.3 mg/kg) 45 minutes before exposure then with saline or the 5-HT4 receptor agonist (RS 67333, 1 mg/kg), 30 minutes before exposure to the sample trial of the object recognition task. Immediately after the testing trial, both particulate (white bar) and soluble (black bar) fractions from the hippocampus (**a**), the prefrontal cortex (**b**) and perirhinal cortex (**c**) were isolated and were assayed for PP2 activity. PP2 activities were pmol of phosphate released by min and mg protein. Results are means ± SEM of four independent subcellular fractionations performed in triplicate. Within each subcellular compartment, # indicated significant differences of PP2 activity as compared with other treatment (#, P<0.05, ##, P<0.01, ###, P<0.001, ANOVA followed by Fisher's LSD test).

We assessed PP2 activities in both subcellular compartments (soluble and particulate) of the hippocampus, prefrontal and perirhinal cortices since excessive cAMP catabolism is limited by reversing phosphorylation of particulate PDE4 activities [53]–[55]. PP2 activity was measured in both the soluble and particulate fraction (**Table 4**); PP2 activity was however mainly present in the soluble fraction (68.1±2.1% to 75.3±1.7% of total PP2 activity according to the structure considered). Milrinone significantly altered PP2 activities in the soluble fraction of the hippocampus (**Fig. 7a**) and perirhinal cortex (**Fig. 7c**) as well as the soluble and particulate fractions of the prefrontal cortex (**Fig. 7b**). In fact, a slight decrease was evidenced in the soluble fraction of the hippocampus from RS 67333-treated rats (−20%, P<0.01, **Fig. 7a**). Thus, in milrinone-treated rats, soluble PP2 activities decrease (−44% when compared to saline rats, P<0.001) was significantly strengthened (P<0.01) when rats were also injected with RS 67333 (PP2 activities was diminished by −70% when compared to saline rats, P<0.001). Similar observations were done in the prefrontal cortex of milrinone-treated rats (**Fig. 7b**), in which PP2 activities decrease in the soluble fraction (−53% when compared to saline rats, P<0.001) was significantly strengthened (P<0.01) when rats were also injected with RS 67333 (PP2 activities was diminished by −79% when compared to saline rats, P<0.001). PP2 activities of the particulate compartment were altered in milrinone-treated rat (−79%, P<0.001) but this decrease was not strengthened in milrinone+RS 67333 treated animals. Finally, milrinone affected soluble PP2 activities in the perirhinal cortex, when administrated alone or in association with RS 67333 (−75% when compared to saline-treated rats, P<0.001; **Fig. 7c**).

**Table 4:** Distribution of the total PP2 activities between particulate and soluble fractions from rat hippocampus, prefrontal cortex and perirhinal cortex.

	Total PP2 activities (nmol of phosphate released/min) (% of the total PP2 activities)		
	Hippocampus (n = 4)	Prefrontal cortex (n = 4)	Perirhinal cortex (n = 4)
**Particulate fraction**	15.83±1.18 = 24.7±1.7%	2.58±0.24 = 32.0±2.1%	3.38±0.39 = 27.8±1.7%
**Soluble fraction**	48.07±0.9§§§ = 75.3±1.7%	5.47±0.27§§§ = 68.1±2.1%	8.73±0.20§§§ = 72.2±1.7%

Values are means ± SEM.

§§§ *P*<0.001 (ANOVA followed by Fisher's LSD test): different from the corresponding particulate fraction.

Such modifications of PP2 activities could not be evidenced in rolipram-treated animals (data not shown).

## Discussion

Object recognition memory in rodents and primates is thought to be mediated, at least in part, by interactions between the perirhinal cortex, prefrontal cortex and hippocampus [66]–[70]. Interestingly, 5-HT4R, known to be involved in learning and memory [31], including acquisition of information [18] are widely distributed among these brain structures [71]–[73]. Since 5-HT4R stimulation induces an increase in cAMP that has been associated to memory processes [6], [8], [9], [11], we first hypothesized that the drug-induced increase in cAMP might support the improvement of object recognition memory performance. Data presented here strongly suggest that the “pre-sample” early increase in cAMP levels followed by a specific lowering of cAMP concentrations in each brain sub-region involved to the object recognition paradigm improve learning efficacy after a middle-term delay.

We first confirmed the major role of PDE3 and PDE4 in the control of cAMP levels in the anatomical structures linked to the object recognition task [18]. Indeed, we reported that both the stimulation of cAMP production (RS 67333) and the inhibition of its hydrolysis (milrinone, PDE3 inhibitor or rolipram, PDE4 inhibitor) in the rat, improve familiar object recognition after a 4-h delay. Besides, we observed similar effects of PDE3 and PDE4 inhibitors. In line with previous studies, we reported a higher sensitivity of PDE3- than PDE4-regulated cAMP pools to PKA activation, associated to a lower efficacy of PDE3 to hydrolyze cAMP [74]–[77]. We hypothesized this familiar object recognition improvement to be linked to the early cAMP levels increase measured before the sample phase in the hippocampus, prefrontal and perirhinal cortices from these animals. Early cAMP activations in the prefrontal cortex have already been described to be beneficial for working memory under conditions that require hippocampal-prefrontal cortex interactions [14], [78]. Moreover, the observation that the drug-induced early increase in cAMP in the brain sub-regions improves the mnesic trace is consistent with data reporting that activation of the cAMP-PKA pathway cascade improves memory processes [2], [4], [6], [8], [9] unlike inhibition of PKA [6], [10], [11], [79]. Indeed, according to the model of Frankland and Bontempi, experience is both initially encoded in hippocampal and cortical networks. Subsequent reactivation of the hippocampal network reinstates activity in different cortical networks. This coordinated replay across hippocampal–cortical networks leads to gradual strengthening of cortico-cortical connections, progressively disengaging the memory trace from the hippocampus [80]. Thus, the higher cAMP levels induced by drug treatments could support a better acquisition of the mnesic trace that will, in turn, benefit to the animal during the testing phase.

We also observed that drug treatments induce a lowering, and/or a reduction of the awaited increases, in cAMP levels in all brains regions studied after the testing phase, when compared to saline injected animals. Hence, cAMP concentrations were systematically lower in both the hippocampus and perirhinal cortex from animals that have increased behavioural performances than in the saline-treated rats that exhibit poor object recognition performances. Appearing at a first glance as a discrepancy from data from the literature, this situation can easily be reconciled by recent studies suggesting that memory requires a restricted, or selective cAMP production rather than a large and widespread increase in cAMP levels [28], [81]. Indeed, Kelly and co-workers (2008) observed an impairment of memory consolidation and/or retrieval in a fear-conditioning task in mice that express a constitutively active isoform of the G-protein subunit Gαs in the forebrain [81]. Perez-Garcia and Meneses, (2008) also demonstrate that the hippocampal production of cAMP was higher in untrained rats than in rats subjected to a behavioral task [28]. In this respect, low cAMP levels might be optimal to convert temporary memory during acquisition to long-term memory (4 h-delay) while high cAMP levels might disturb such a conversion of short-term memory to long-term memory, resulting in low performances in controls animals.

These few elements point out the complexity of cAMP-dependent responses and the putative interactions between behaviour- and drug-induced effects on cellular signaling. By rapidly degrading cAMP from selected compartments, PDEs can fix the boundaries for cAMP diffusion, shape the intracellular gradients of the second messenger and thereby modulate defined sets of PKA-mediated intracellular events. Hence, PDE alteration may affect cAMP compartmentalization, leading to untargeted cAMP signals, aberrant phosphorylation of target proteins and thus contribute to dysfunction. We report here that hippocampus, prefrontal and perirhinal cortices exhibit different patterns of particulate PDE4D isoforms. One can therefore hypothesize differential implications of PDE isoforms, keys mediators of memory and learning processes in the limitation of cAMP increase [36], [37], [82], [83]. For example, PDE4D8 found only in the particulate fraction from the hippocampus or the prefrontal cortex, has been shown to be responsible for controlling local cAMP concentrations and PKA activity in the vicinity of β1 adrenergic receptors [46]. PDE4D3 that we only described in the particulate fraction from the hippocampus or the prefrontal cortex, was reported to bind to muscle- specific A-kinase anchoring protein (mAKAP), which in turns controls perinuclear AMP levels and recruits the MAP kinases MEK5 and ERK5 [84]. Hence, each PDE isoform plays a critical role in the specificity of cAMP-signaling, effectively creating cyclic nucleotide microdomains and/or cAMP gradients that can be sensed by the cell [46], [85]–[88]. Accordingly, some PDE4D isoforms precisely regulate the coupling between GPCR and the Gs protein. A desensitization of 5-HT4R, through an over-stimulation by RS67333 and/or an alteration of PDE, could thus lead to a lowering of cAMP concentrations in brain regions expressing 5-HT4R. Indeed, PKA activated after a 5-HT4R stimulation, will phosphorylate 5-HT4R leading to cell membrane recruitment of GRK2, which in turn will phosphorylate i) the associated GPCR [89] inhibiting its coupling with Gs, and ii) PDE4, which locally attenuates the PKA activity by lowering local cAMP levels [90]–[92].

Effects induced by 5-HT4R stimulation are time-limited since PKA phosphorylation of 5-HT4R, GRK2 or PDE can be reversed by phosphatase (PP) activity [58]. PP1 and PP2, compartmentalized inside the mammalian cell [93], [94], account for the major phosphatase activities [56]; PP2 is however more particularly investigated because of its ability to dephosphorylate many signaling proteins [93]. Since PP2A is activated by cAMP level increases [53]–[55], we suggested that phosphatase activity may have been raised in the brain sub-region structures from drug-treated rats, but it is not what we found here. While inhibition of PDE4 activity failed to alter PP2 activity, milrinone administration induced an alteration of PP2 activity, especially in the supernatant fractions from the brain regions investigated; such an alteration could in turn alter dephosphorylation of the 5-HT4R, prevent the efficiency of coupling between this receptor and the Gs protein, and thus lead to a lowered cAMP production. Such a functional contrast following selective inhibition of PDE3 and PDE4 has been already observed in many cell types [95]–[97]. Interestingly, the cell membrane recruited PDE4 also desensitizes the switched coupling of the β2AR to activation of Gi induced by the PKA-mediated phosphorylation [98], defining thus an appropriate coupling of the GPCR [99]. Hence, in our opinion, drugs injected before the sample phase rapidly increase cAMP levels leading to the uncoupling of 5-HT4R. However, during the test phase, further 5-HT4R stimulation does not raise cAMP level, probably because of 5-HT4R uncoupling. 5-HT4R uncoupling could thus be an adaptative mechanism to reduce cAMP levels in the presence of an excessive stimulation of 5-HT4R or an absence of PDE3 or 4 activities thus avoiding an excessive accumulation of cAMP. If such threshold of cAMP level exists and is reached by either the stimulation of 5-HT4R or inhibition of PDE3 or PDE4 alone, thus no further improvement of memory performance could be induced by the pharmacological treatments by the combination of RS 67 333 and PDE inhibitor.

Finally, early increases in cAMP levels followed by an immediate drop in cAMP concentrations have already been well described in cell differentiation, particularly in Sertoli cells [58]. Indeed, before the cAMP increase, stimulation of Sertoli cells by gonadotropin leads to an activation of the ERK pathway, while following the peak of cAMP, gonadotropin activates the PKA pathway. Interestingly, ERK pathway could prolong activation of the cAMP signaling system in cells by having both short and long term effects on PDE4D activity by respectively inactivating long PDE4D isoform (the ones to exhibits a site that allows phosphorylation by ERK) and altering PDE4D mRNA stability (for review [99]). Hence, by analogy to differentiation mechanisms, another hypothesis is that “cellular learning” may result from the crossing of a milestone, resulting in the subsequent activation of alternative intracellular signalling pathways. Increases in cAMP levels but also their subsequent declines account in mnesic performance improvement. The part of ERK pathway in these processes should be addressed in furthers works.

Our results show that a “pre-sample” early increase in cAMP levels followed by both a “post-sample” lowering of cAMP concentrations in the prefrontal cortex and a “post-test” lowering of cAMP concentrations in the hippocampus and perirhinal cortex support improved learning efficacy after a middle-term delay. If cAMP triggers a temporally defined cellular response, a major question that should be addressed in future works is to clarify how such a functionally ubiquitous signaling pathway may be involved in memory formation.
